# Lipidomics of Huntington’s Disease: A Comprehensive Review of Current Status and Future Directions

**DOI:** 10.3390/metabo15010010

**Published:** 2025-01-02

**Authors:** Ali Yilmaz, Sumeyya Akyol, Nadia Ashrafi, Nazia Saiyed, Onur Turkoglu, Stewart F. Graham

**Affiliations:** 1 Department of Obstetrics and Gynecology, Oakland University-William Beaumont School of Medicine, Rochester, MI 48309, USA; ali.yilmaz@corewellhealth.org (A.Y.); nadia.ashrafi@corewellhealth.org (N.A.); onur.turkoglu@bcm.edu (O.T.); 2 Metabolomics Division, Beaumont Research Institute, 3811 W. 13 Mile Road, Royal Oak, MI 48073, USA; nazia.saiyed@corewellhealth.org; 3 NX Prenatal Inc., 4350 Brownsboro Rd, Louisville, KY 40207, USA; sakyol@nxprenatal.com

**Keywords:** Huntington’s disease, lipidomics, biomarker discovery, NMR, mass spectrometry

## Abstract

Background: Huntington’s disease (HD) is a multifaceted neurological disorder characterized by the progressive deterioration of motor, cognitive, and psychiatric functions. Despite a limited understanding of its pathogenesis, research has implicated abnormal trinucleotide cytosine-adenine-guanine CAG repeat expansion in the huntingtin gene (HTT) as a critical factor. The development of innovative strategies is imperative for the early detection of predictive biomarkers, enabling timely intervention and mitigating irreversible cellular damage. Lipidomics, a comprehensive analytical approach, has emerged as an indispensable tool for systematically characterizing lipid profiles and elucidating their role in disease pathology. Method: A MedLine search was performed to identify studies that use lipidomics for the characterization of HD. Search terms included “Huntington disease”; “lipidomics”; “biomarker discovery”; “NMR”; and “Mass spectrometry”. Results: This review highlights the significance of lipidomics in HD diagnosis and treatment, exploring changes in brain lipids and their functions. Recent breakthroughs in analytical techniques, particularly mass spectrometry and NMR spectroscopy, have revolutionized brain lipidomics research, enabling researchers to gain deeper insights into the complex lipidome of the brain. Conclusions: A comprehensive understanding of the broad spectrum of lipidomics alterations in HD is vital for precise diagnostic evaluation and effective disease management. The integration of lipidomics with artificial intelligence and interdisciplinary collaboration holds promise for addressing the clinical variability of HD.

## 1. Introduction

Huntington’s disease (HD) is a rare, genetically inherited neurological condition caused by a CAG triplet repeat expansion in the huntingtin gene (HTT) encoding the huntingtin protein (htt) [1]. It is characterized by progressive damage to the striatum, unwanted choreatic movements, behavioral and psychiatric issues, and cognitive decline [2,3,4]. Huntingtin (HTT) is a large, 348 kDa protein that plays a vital role in embryonic development and participates in a wide range of cellular activities, including vesicular transport, endocytosis, autophagy, and transcriptional regulation [5,6]. While the precise biological functions of HTT are not yet fully understood, the large number of proteins that interact with HTT suggest that it plays a key role in facilitating protein–protein interactions. This hub-like function may be crucial for coordinating various cellular processes [5,6,7]. However, only limited structural information regarding HTT is currently available. A recent study by Guo et al. focused on the determination of the structure of full-length human HTT in a complex with HTT-associated protein 40 (HAP40, encoded by three F8A genes in humans) via cryo-electron microscopy to an overall resolution of 4 Å [8]. It was reported that that HTT is primarily composed of α-helices, organized into three major domains and a smaller bridge domain. The three major domains contain HEAT (huntingtin, elongation factor 3, protein phosphatase 2A, and lipid kinase TOR) repeats arranged in a solenoid fashion [9]. These domains are connected by a smaller bridge domain containing different types of tandem repeats. HAP40 is also largely α-helical and has a tetratricopeptide repeat-like organization. HAP40 binds in a cleft and contacts the three HTT domains by hydrophobic and electrostatic interactions, thereby stabilizing the conformation of HTT [10,11]. The onset, severity, and progression of HD vary widely among individuals, influenced by factors such as CAG repeat length, genetics, and environment [12]. Typically, symptoms emerge between ages 30 and 50, but in some cases, they may appear before age 20 [13]. Individuals with HD typically have a CAG repeat length between 36 and 250, although some people with a repeat length below 42 may not exhibit symptoms [14]. HD occurs with a frequency of approximately 1 in 10,000 individuals in most populations of Caucasian descent [15], and appears to be more prevalent in people of European, North American, and Australian ancestry compared to those of Asian background. Children of parents with HD have a 50% chance of inheriting the condition. To date, studies have mainly focused on mitigating symptoms rather than on determining how to halt, or even reverse progression [15,16]. Despite the identification of the responsible gene, HTT, 30 years ago, there is currently no cure for HD, emphasizing the need for reliable biomarkers to track disease progression and evaluate therapeutic effectiveness. This will help advance disease-modifying strategies and deepen our understanding of HD’s underlying mechanisms [17]. HD is a monogenic Mendelian disorder. Therefore, the pathogenesis of the disease is molecular rather than environmental. The familial nature of HD necessitates a comprehensive approach to identifying molecular biomarkers, one that includes a thorough examination of the extended family, recognizing that the risk of inheritance is significantly higher among family members than in the broader population. The pathophysiology of HD is distinguished by a multifaceted interplay of molecular alterations and clinical manifestations, which emerge and evolve across the lifespan as a direct consequence of the underlying genetic mutation. Accurately defining an HD biomarker requires collecting comprehensive data to uncover the disease’s underlying mechanisms.

Biomarker studies, including clinical, imaging, and biofluid research, have shed light on the key processes driving HD pathogenesis. It has been demonstrated that impaired proteasome activity [18], transcriptional dysregulation [19], oxidative stress [20], mitochondrial and metabolic dysfunction [21], neuroinflammation [22,23], and microglial activation [24,25,26] play significant roles in the pathogenesis of HD.

Lipids are a structural and functionally diverse group of biomolecules, typically featuring an alkyl chain with a carboxyl group; they encompass various categories, including fatty acyls, glycerolipids, and sphingolipids [27]. Lipids are a diverse group of biomolecules comprising fatty acyls, glycerophospholipids (GP), sphingolipids (SP), sterol lipids (SL), and glycerolipids (GL); they serve as the primary building blocks of biological membranes [27,28]. The LIPID MAPS consortium introduced a comprehensive lipid classification scheme in 2005 that categorizes individual lipid molecular species into eight distinct categories [29]. In each category, individual lipid molecular species are further divided into lipid classes based on their polar head groups. For example, glycerophospholipids are grouped into the classes of PC, PE, PI, serine glycerophospholipid (PS), etc., according to whether their polar head groups contain phosphocholine, phosphoethanolamine, phosphoinositol, phosphoserine, or others, respectively, linked to a glycerol backbone [30]. These lipids are essential for maintaining the structural and functional integrity of membranes, storing energy, and facilitating communication between cells [31,32].

Brain cells, including neurons, oligodendrocytes, glial cells, astrocytes, and others, are protected against fluctuations in chemical or ion concentration because they are isolated from other parts of the body through the blood–brain barrier (BBB). Cellular membranes are essential for preserving brain chemical and ion balance. Therefore, disruptions in lipid composition and distribution within cell membranes can have severe pathological consequences, impacting essential physiological processes like ion channel function and receptor activity [33]. Cells must synthesize and transport new lipid components to the plasma membrane during the secretion of neurotransmitters, hormones, and enzymes. The phospholipid component of the plasma membrane is continually replenished by lipase and acyltransferase enzymatic activity [34,35]. Obviously, lipids play a crucial role in maintaining the structure and function of the central nervous system (CNS), and disruptions in lipid metabolism are associated with various neurological disorders, including HD. As the brain is the second most lipid-rich organ, lipidome analysis a valuable tool for understanding brain function, ageing, and neurological disease mechanisms [36]. The discovery of direct interactions between htt and membrane lipids implies that alterations in lipid composition may contribute to the neuronal damage and dysfunction characteristics of HD [37].

Lipidomics represents a combination of systems biology and emerging multidisciplinary approaches, synergically integrating lipid biology, innovation in bioanalytical techniques, and medical science to construct a comprehensive map of the lipidome, i.e., the entire lipid pool within a cell or tissue [38]. The primary objective of lipidomics research is to identify the lipid metabolic pathways and networks affected by various perturbations, such as disease states, genetic mutations, therapeutic treatments, or other factors. Due to their hydrophobic properties, lipids need to be treated and analyzed separately (i.e., requiring different solvent systems) from small-molecule metabolites [39]. Although initially considered a subset of metabolomics, lipidomics has emerged as a distinct field of research due to the remarkable structural and functional diversity of lipids and their enormous endogenous abundance.

This review will cover the roles of lipids in brain physiology as well as their relationship to disease progression, and summarize lipidomic studies in humans, animals, and biological fluids using various analytical techniques (Table 1), focusing on HD.

## 2. Lipid Constituents of the Brain

**Figure 1:**
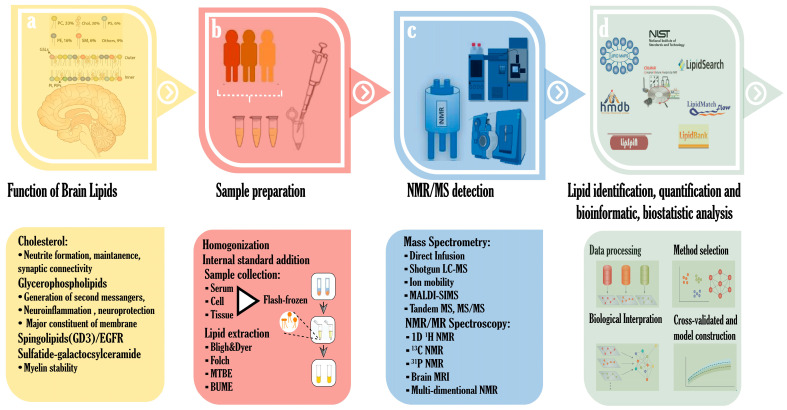
Important components of lipidomics research on HD: lipid composition of the brain and lipid roles in the brain (**a**); sample collection and important analytical techniques for lipid extraction (**b**); main analytical platform currently in use for lipidomics research (**c**); available lipid databases available for NMR- and MS-based lipid identification and tools for bioinformatic and biostatistical analysis (**d**).

Notably, following adipose tissue, the brain is the second most lipid-dense human tissue, with lipids making up around 50% of its dry weight [64]. The three main components of the brain are cholesterol, phospholipids—including phosphatidylethanolamine (PE) and phosphatidylcholine (PC)—and sphingolipids (Figure 1a) [65]. A notable characteristic of the brain is its exceptionally high cholesterol content, surpassing that of other organs and biological fluids. It comprises approximately 25% of the body’s total cholesterol content. Through the production of cholesterol-rich lipoproteins, such as apolipoprotein E (APOE), lipids are transported to neurons [66]. In neurons, cholesterol is utilized for neurite maintenance and synaptic connectivity [65,66]. Also, myelin sheaths in the brain are where most of the endogenous cholesterol is located [67]. As lipid droplets, which store additional cholesterol inside cells, around 1% of the cholesterol in the brain is esterified [67]. It should be noted that, compared to other tissues, the brain has the highest concentration levels of unesterified cholesterol. The BBB plays a crucial role in maintaining the brain’s sterol homeostasis by severely limiting the entry of sterols from the bloodstream. As a result, the majority of sterols present in the CNS are in situ [68,69]. Maintaining normal cholesterol homeostasis is vital, as disturbance can lead to degenerative disease and cognitive decline [70,71].

Glycerophospholipids, essential components of biological membranes, play a crucial role in neuronal membrane function and fluidity. The most abundant types, PC and PE, are synthesized via the Kennedy pathway [72,73]. Phospholipases break down glycerophospholipids, generating second messengers like arachidonic acid and long-chain polyunsaturated fatty acids (LC-PUFAs), which are involved in neuroprotection and anti-inflammation. Acute brain conditions, such as ischemia and trauma, alter glycerophospholipid composition and enzyme activity, highlighting their significance in neural membrane function and response to injury [72,74,75,76].

Sphingolipids, crucial membrane components, comprise a sphingosine backbone linked to a single fatty acid [77]. They play key roles in regulating cell growth and differentiation, maintaining brain function (including neurogenesis and synaptogenesis), interacting with transmembrane proteins in lipid rafts and cholesterol, and modulating neurotransmitter receptors in synaptic membranes [78,79,80,81]. Sphingolipids play distinct roles depending on their localization within cellular membranes. In lipid rafts, sphingolipids interact with cholesterol to regulate the activity of transmembrane proteins. In contrast, the sphingolipids present in synaptic membranes interact with neurotransmitter receptors, thereby modulating their activity and influencing neurotransmission [82,83]. The sphingolipid family includes sphingomyelin, gangliosides, cerebrosides, and sulfatides, all derived from ceramides (N-acetylsphingosine) [65,84].

Ceramides undergo further modifications, including the addition of branching and hydroxyl groups, leading to the formation of various sphingolipids, such as sphingomyelin, cerebrosides, and glycosphingolipids [85]. The production of ceramides takes place in the endoplasmic reticulum of both neurons and glial cells, and they are then transported to the trans-Golgi regions of these cells by ceramide transporter proteins, also known as CERTs. These CERTs are expressed throughout the central nervous system and play a crucial role in brain development and maintaining homeostasis [86,87,88,89].

The brain’s white matter contains a high concentration of sphingomyelin (SM) and its metabolites, including cerebroside and sulfatide, which are essential constituents of the myelin sheath. This sheath facilitates nerve impulse transmission by acting as an insulator and regulating the saltatory conduction mode through the nodes of Ranvier [90]. Gangliosides, a distinct class of glycosphingolipids, are characterized by a ceramide backbone and a glycan headgroup containing sialic acid residues. These molecules are particularly abundant in the central nervous system, including the brain, where they play a crucial role in modulating cell signaling pathways and conferring neuroprotection. Fatty acids (FAs) play a vital role in the brain, where they serve multiple purposes. These include acting as signaling molecules, providing energy, and serving as precursors for the synthesis of essential phospholipids and sphingolipids. Furthermore, astrocytes, a type of glial cell, are responsible for oxidizing FAs to produce energy. This process is crucial, as it meets approximately 20% of the brain’s energy demand [86,91].

## 3. Instrumentation and Recent Advancement in Lipidomics

Lipidomics, like other omics fields, is a dynamic discipline driven by technological advancements. It continuously integrates new developments in analytical chemistry, including techniques, instruments, statistical methods, software, and computational tools to enhance data acquisition, processing, and interpretation [92]. The extraction of lipids from brain tissue, cells, and various biological fluids, such as serum, plasma, and cerebrospinal fluid (CSF), can be efficiently performed using liquid–liquid extraction techniques, including the Folch method, Bligh and Dyer method, and alternative approaches employing methyl tert-butyl ether and n-butanol/methanol [93,94]. (Figure 1b). To facilitate the MS-based quantification of diverse lipids, internal standards from various lipid classes are typically added to the sample prior to extraction. Optionally, the lipid mixture can be separated into polar and nonpolar components to simplify molecular complexity and enable efficient profiling. The use of varied organic solvent and water ratios aids in extracting and concentrating brain lipids, supporting comprehensive profiling [93]. In lipidomics research, three primary analytical platforms have been employed: nuclear magnetic resonance (NMR) spectrometry, the fluorescence assay, and mass spectrometry (MS) (Figure 1c) [95]. Each platform has its strengths and weaknesses in terms of performance, sensitivity, and efficiency, and the choice of platform depends on the study’s objectives and sample characteristics [96]. While fluorescence assays are the simplest method for measuring specific lipid components, they are unsuitable for comprehensive lipid profiling due to their limitations [97,98]. NMR spectroscopy is gaining popularity in lipidomics due to its reproducibility, high resolution for molecular structure elucidation, and non-destructive nature [99]. However, its inherent low sensitivity remains a drawback [100]. In contrast, MS-based techniques offer high sensitivity, detecting lipid analytes at nanomolar or picomolar concentrations, but may compromise reproducibility [101,102].

Mass spectrometry (MS)-based techniques are the most advanced and powerful tools for analyzing cellular lipid metabolic networks and their synthesis in biological systems [103]. Mass spectrometry is a sensitive tool that allows the identification and quantification of various classes, subclasses, and individual molecular species of lipids in a biological sample at the nanomolar and even picomolar level [92,104]. However, MS-based lipidomics faces limitations due to differences in ionization efficiency, signal intensity, and ion-quenching phenomena, in which the signals from poor ionizing lipids are quenched by more readily ionized species, leading to reduced sensitivity for non-polar lipid metabolites [99]. To address this, researchers employ either untargeted or targeted methodologies for NMR and MS analyses [105,106]. Untargeted approaches like “shotgun” lipidomics detect unidentified metabolites but require additional validation tests. Targeted approaches, on the other hand, involve the deliberate quantification of specific analytes with well-characterized structures. Currently, the application of MS technology in lipidomics is hindered by a lack of standardization across the various techniques used, including liquid chromatography (LC)–triple quadrupole, LC–quadrupole time-of-flight, LC–Orbitrap, nano-electrospray ionization (nano-ESI), gas chromatography (GC-MS), acoustic ejection, and matrix-assisted laser desorption ionization–time of flight (MALDI-TOF), each with its advantages and limitations (Table 2).

Recent advancements in instrumentation have led to the adoption of techniques like shotgun MS, LC-MS, and GC-MS in lipidomics research [107,108]. Cutting-edge MS technology offers high sensitivity and resolution, enabling the in-depth analysis of diverse lipids, including isomers [103]. However, LC- and GC-MS methods require complex sample preparation, potentially reducing lipid recovery [109]. Furthermore, molecular separation increases analysis time, leading to inter-batch variations when processing large sample batches. Despite these challenges, determining the sn-positions of acyl chains and confirming the position and conformation of carbon–carbon double bonds remains difficult. Nevertheless, ongoing research is expanding the capabilities of traditional lipid characterization with innovative methods like the Paterno–Büchi reaction, ozonolysis, ultraviolet photodissociation, and the electron impact excitation of ions from organics [110,111]. Matrix-assisted laser desorption ionization (MALDI) and mass spectrometry imaging (MSI) represent valuable analytical techniques for the elucidation of the spatial distribution of specific brain lipids, thereby providing a complementary approach to MS systems relying on chromatographic separation [112,113].

This approach enables the creation of a molecular–histological map of the brain, revealing the regional distribution of lipids. In MALDI-MSI, frozen brain tissue is sectioned, covered with a matrix, and analyzed using direct MALDI–time-of-flight (TOF) MS or TOF-MS. Although MSI excels in spatial analysis, quantifying lipid species remains challenging. Lipid extraction from brain tissues, cells, and biofluids like serum, plasma, and CSF employs techniques such as Folch, Bligh and Dyer, methyl tert-butyl ether, or n-butanol/methanol methods. Internal standards are added to the sample before extraction for the MS-based quantification of diverse lipid structures. To simplify molecular complexity, the lipid mixture may be fractionated into polar and nonpolar components [59]. Various organic solvent and water ratios are used to extract and enrich brain lipids, facilitating profiling. In MS-based lipidomics, triple-quadrupole (QQQ)–MS is the most widely used technique for the targeted quantitative analysis of specific lipids, potential biomarkers, or markers involved in particular lipid pathways. Although QQQ-MS is not suitable for comprehensive lipidome analysis, its multiple reaction monitoring (MRM) mode excels in quantifying targeted lipid species related to neurodegenerative disorders [114]. In contrast, untargeted lipidomic analysis aims to extensively characterize lipidomes using high-resolution tools like quadrupole–TOF or Orbitraps. Untargeted lipid analysis has been performed using shotgun MS, LC-MS, and GC-MS. Recently, ion mobility–MS has been employed to investigate multiple lipids from brain cells and specific locations, providing high-accuracy collision cross-sectional information and increasing lipid identification confidence [115,116]. Focusing on lipids, NMR spectroscopy is an invaluable tool for investigating the lipid structure and dynamics of functioning cells due to its non-destructive nature.

Most recently, ^1^H NMR-based lipidomic analyses of high-density lipoproteins have proven NMR is particularly useful for characterizing of the atheroprotective function of high-density lipoproteins, uncovering molecular insights and characterization, identifying novel biomarkers of disease, and monitoring therapeutic interventions [117].

NMR spectroscopy-based lipid analysis techniques often face challenges such as signal overlap in either the ^1^H NMR or ^31^P NMR spectrum and low sensitivity due to the low natural abundance of the ^13^C isotope. To address the limitations of conventional NMR spectroscopy, various hyperpolarization techniques have been established, including the optical pumping of ^3^He or ^129^Xe, parahydrogen-induced polarization (PHIP), and dynamic nuclear polarization (DNP), which collectively enable substantial enhancements in NMR sensitivity. Of these, PHIP and DNP have emerged as promising hyperpolarization techniques for lipidomics applications [118,119]. Nonetheless, ^13^C NMR offers valuable complementary information compared to conventional ^1^H NMR. Particularly, it allows for the clear resonance of the carbon atoms in the glycerol backbone, aiding in the determination of glyceride and partial glyceride compositions. Additionally, even though ^13^C NMR measurements are constrained in lipidomic research, they have the capability to differentiate carboxylic acid groups from other carbonyls. These advantages have applications in studying lipase specificity and detecting abnormal lipid metabolism [120,121]. The Metabolomics Workbench, established by the NIH Metabolomics Program, is a premier online platform providing access to a vast database of metabolites, including lipids. This database boasts an impressive collection of approximately 60,000 unique metabolite species [92]. The structural data for these species were compiled from sources like LIPID MAPS, the human metabolome database, and the Kyoto Encyclopedia of Genes and Genomes.

Concurrent with the progress in NMR spectroscopy and MS-based lipidomics, the development of lipid databases (DBs) and software tools has enabled the efficient management of large-scale datasets, facilitating the compositional and structural profiling of multiple lipids, as well as the analysis of their molecular interactions (Figure 1d). The largest lipid-only database, LIPID MAPS, contains structural information on nearly 40,000 lipid species [122]. Now, the LIPID MAPS website serves as a comprehensive platform facilitating the interpretation of MS data, with a focus on the interpretation and statistical analysis of biologically relevant lipids. Other lipidomics-focused databases include lipidr and LipidSig. Table 3 lists the DBs that are accessible for metabolomics (including lipidomics) and just for lipidomics. The NIH Metabolomics Program’s Metabolomics Workbench is a prominent portal for metabolites, including lipids, with around 60,000 species in its database [123]. The structural data for these lipid species were compiled from sources like LIPID MAPS, the human metabolome database, and the Kyoto Encyclopedia of Genes and Genomes. The Metabolomics Workbench facilitates the integration, analysis, and depletion of diverse data from lipidomics experiments using MS and NMR, streamlining research workflows [123]. The availability of reference data for lipidomic research has increased because of all these improvements in NMR- and MS-based lipidomics and software tools for data interpretation, which have facilitated large-scale investigations. Recently, brain lipidomics has leveraged these analytical methods and databases, highlighting the need for a comprehensive and in-depth characterization of brain lipids, as many remain unidentified.

One should be aware that the exponential growth of lipidomics biological data has created a significant challenge in interpreting and extracting meaningful insights. However, the integration of artificial intelligence (AI) in bioinformatics has emerged as a powerful tool for unravelling complex multidimensional datasets [124]. By harnessing AI to analyze lipidomics data, researchers can leverage a robust predictive approach to improve the identification of diagnostic biomarkers, integrate clinical data, and investigate neural behavior, novel etiology markers for inflammation, and the progression of neurodegeneration. Additionally, AI can aid in mass spectrometry image analysis, potentially leading to the discovery of novel biomarkers and molecular pathways related to lipid dysfunction in HD [124]. Lipidomics plays a vital role in HD research by connecting the disease’s phenotype and genotype, mirroring alterations in the genome, transcriptome, and proteome. Integrating brain omics research with AI-based platforms will facilitate large-scale cohort studies and long-term follow-ups, bridging the gap between the phenotype and genotype linked to the disease. This synergy will enable the development of preventive treatments for HD, addressing its complex biological heterogeneity and dynamic progression. By combining omics research with AI, researchers can tackle HD’s intricate mechanisms, leading to more effective prevention and treatment strategies.

## 4. Animal Models

In the initial stages of HD research, investigators employed toxin-induced models to investigate the two separate mechanisms responsible for the deterioration observed in the brains of individuals with HD. These mechanisms involve cellular death triggered by excitotoxicity and damage to the mitochondria. For example, substances such as quinolinic acid and 3-nitropropionate were administered to mice for this purpose [125]. After the identification of htt, animal models with similar genetic abnormalities were introduced. These knock-in and transgenic mouse models provide a more accurate representation of HD pathology and its progression. Advances in molecular technologies have enabled the development of genetically modified animal models, such as mice and rats, to mimic the genetic characteristics of HD. These animal models were created by introducing mutant-htt (M-htt) proteins into the germline genes of rodents, resulting in strains with either full-length or truncated versions of the mutated HTT (M-HTT). These genetic modifications were introduced through either random genomic integration or targeted insertion into the HTT locus, allowing for a more accurate representation of the disease in animal models. To mimic the genetic characteristics of HD, varying repeats of CAG are important to provide insight into disease metabolism. The earliest and first transgenic mouse models included R6/1, expressing 114 CAG repeats, and R6/2, expressing 150 CAG repeats. These mice manifested pronounced and early behavioral and anatomical symptoms characteristic of HD and carried the human HTT (notably, a segment of the mutant exon 1). Additionally, N171-82Q expresses a truncated version of M-htt with 82 CAG repeats, showing prolonged symptoms compared to R6/2. Additionally, artificial yeast chromosome (YAC1) technology has been used to create transgenic mouse and rat models, with the latter exhibiting more complex behaviors and a longer lifespan, making it a valuable resource for investigating long-term treatments [126]. In this model, the duration of both the behavioral and anatomical symptoms is prolonged compared to the R6/2 model. One method of generating an artificial yeast chromosome (YAC1) involves the cloning of an artificial yeast vector containing extended polyglutamine sequences directly into the mouse genome [127]. The approach was also applied in the development of a transgenic rat model [97], wherein the rats manifested severe anatomical and behavioral impairments and expressed the CAG repeat sequence 51 times. This transgenic rat model generally exhibits a lifespan over one year longer than that of mice. Due to the more intricate behavioral patterns of rats, this transgenic rat model is regarded as a more effective resource for investigating long-term treatments for HD, as highlighted in reference [128]. Genetic modifications can be made to specific brain regions using viral vectors, but due to the difficulties in creating genetic models in rats, nonhuman primates are often a more efficient option [125]. Various animal models present distinct advantages for the study of HD. Large animal models, in particular, are advantageous due to their similarities to humans in metabolism, brain and spinal cord size, immune system complexity, and longer lifespan. These models enable extended longitudinal studies, allowing for the exploration of new treatments and providing valuable insights into the disease’s etiology and prevention, as they closely mimic human pathophysiology [129,130].

In this regard, the HD sheep model provides a unique benefit: the ability to track metabolic changes over an extended period, enabling researchers to monitor disease progression through metabolic alterations and rate markers [95]. The OVT73 sheep model, featuring 73 repeated CAG units of human cDNA transgenes, facilitates this long-term monitoring. Interestingly, these sheep do not display noticeable behavioral changes or brain structural modifications [131,132]. Only subtle neuropathological manifestations [133] and limited postmortem (PM) shifts in cerebellar and liver metabolism [134] are observed, suggesting that even at 5 years of age, the sheep are still in the pre-symptomatic phase of Huntington’s disease. Although HD mouse models, such as R6/2, have been useful in assessing neuroprotective agents like mitochondrial coenzyme Q10 (CoQ10), especially when combined with remacemide [135], clinical trials even at significantly high dosages of CoQ10 have not replicated these results [136,137]. Similarly, creatine trials in early-stage patients were unsuccessful, contradicting the promising preclinical results in R6/2 mice [138,139,140]. These therapeutic failures raise concerns about the reliability of high-definition animal models in predicting human responses, highlighting the need for re-evaluation. The assessment of HD models is critical, particularly considering the limitations of the Unified Huntington’s Disease Rating Scale (UHDRS) and metabolic biomarkers. The UHDRS evaluates four clinical domains, but biomarker performance is compromised by variability and slow MRI response times, highlighting the need for a dual approach to develop more accurate biomarkers [141]. The recent development of the Libechov transgenic minipig model (TgHD) offers promise, as it introduces a human mutant huntingtin exon1 with 124 glutamine repeats (CAGCAA repeat sequence), exhibiting a mild disease phenotype with no early brain aggregation or developmental impairments. This model’s reliable and measurable phenotypes will significantly enhance preclinical testing efficacy, addressing the pressing need for improved biomarkers in HD research [142].

## 5. Application of Lipidomics

### 5.1. Cell Culture and Primitive Models

To elucidate the pathophysiological mechanisms underlying HD and facilitate the development of disease-modifying therapies, researchers have employed lipidomics analysis in clinically relevant cellular models. Using a cell line model of HD, a network-based method called the prize-collecting Steiner forest algorithm [143] for the integrative analysis of untargeted metabolomics (PIUMet) showed that fatty acid, steroid, and sphingolipid metabolism are affected in the disease [41]. To elucidate the role of energy molecules in weight alteration throughout the course of disease progression, Aditi et al. conducted an in vivo transgenic Drosophila model of HD to quantify these models. Notably, the results from previous studies provide evidence supporting the notion that HD is characterized by central alterations in lipid metabolism, which in turn contribute to changes in body weight and a pervasive energy-deficient state [41]. One should be aware that the gradual change in weight that occurs in HD may be caused by the indirect influence of M-htt on the integrated process of lipid homeostasis. Targeting lipid metabolic and/or catabolic pathways through therapeutic approaches may help prevent disease progression and associated weight changes. A lipidomics study by Kegel et al. using COS-1 cells, MCF-7 cells, and X57 cells [144] revealed that polyQ expansion alters the localization of the N terminus of htt (N-htt) to the plasma membrane. Lipid overlay experiments showed no binding differences between wild-type and mutant N-htt fragments, which does not explain the mislocalization of mutant N-htt to the perinuclear region. However, their findings were limited since M-htt may exist in different forms within cells, and the soluble fraction used in the assay may not represent the mislocalized fragments [42].

### 5.2. Application of Lipidomics in Animal Models

Numerous studies using animal models of HD support the hypothesis that lipid metabolism changes occur during disease progression. Microarray studies in HD animal models have identified alterations in mRNA levels that regulate sterol and phospholipid metabolism [43,145]. It was also demonstrated that, compared to wild-type HD, M-htt from mouse brain (Q140/Q140) has more abundant binding and ease of binding to glycerophospholipids in vitro [44]. Furthermore, in pre-symptomatic HD mouse neurons, M-htt accumulates and associates with specific phospholipid-rich membrane fractions, suggesting a link between htt and lipid metabolism [146]. Vodicka et al. performed a lipidomics analysis on glycerophospholipids in Q140/Q140 HD mice at 11 months of age [45]. This approach detected changes in specific species of lysophosphatidic acid (LPA), CL, PC, PG, and PE. In contrast with the wild-type controls, phosphatidic acid (PA) concentrations in the cortex, cerebellum, and striatum of the Q140/Q140 HD mice were significantly different. Specifically, some of the PA species were lower, and four species of LPA were higher. Decreased phosphatidic acid (PA) production may cause a buildup of particular lysophosphatidic acid (LPA) species, such as LPA (14:1, increased; 13:0 m, increased; 17:0 increased; 14:0 m, increased). This is an important activator of G-linked receptors, which can regulate ERK activation, AKT, calcium levels, Rac, and phospholipase C activity, and thereby regulate motor skills and cell morphologic changes, such as proliferation, survival, and neurite retraction [147]. LPA can also activate peroxisome proliferator-activated receptor-gamma (PPARγ) [148]. In one mouse model of HD (C57BL/6 with FVB genetic background), PPARγ was affected, and its activation was protective [149]. These receptors were activated by extracellular LPA, which is produced mainly by lysophosphatidylcholine through the enzyme autotaxin [150]. Because autotaxin is necessary for vascular health [151], changes in LPA might affect the BBB of HD.

In the cerebral cortex, at least seven phosphatidylcholine (PC) species underwent significant changes, potentially due to altered phospholipase enzyme activity or the accumulation of lysophosphatidic acid (LPA) and phosphatidic acid (PA). Notably, an animal model showed elevated levels of specific glycerophospholipids: PA38:4 in the striatum and PE (38:4) and PC (38:4) in the cerebral cortex. These glycerophospholipids, particularly those with arachidonic acid (20:4) at the sn-2 position, can be released by phospholipase A2 (PLA2) and mediate inflammation through leukotriene and prostaglandin production, attracting microglia. Elevated levels of these glycerophospholipids may indicate reduced PLA2 activity. Indeed, the reduction in PLA2 activity is consistent with findings in HD patients who have a reduced niacin-flushing response [46]. Alterations in synaptic function and the integrity of synaptic structure are believed to be early contributors to the cognitive, psychiatric, and motor symptoms of HD. Lately, research by Luliano et al. employed subcellular fractionation to analyze striatal tissue from HD mouse models (Q175/Q7 and Q7/Q7) at 2 and 6 months [4]. The lipidomic analysis revealed significant changes in the levels of key phospholipids involved in synaptic signaling, including PIP2, cholesterol ester, and cardiolipin, in the 6-month-old Q175/Q7 HD mice compared to the wild-type mice. In another study, the biochemical analysis of synaptosomes rich in presynaptic and postsynaptic structures was undertaken using Q140/Q140 HD rats. Notably, the comparison of Q140/Q140 HD rats with WT rats revealed a marked reduction in the levels of C-terminal binding protein 1 (CtBP1) within the striatum synaptosomes of the HD rats.

Local changes in the level of CtBP1 in the synapses could affect the presynaptic levels of PA in HD. Huntingtin protein, which is also present in synapses [152], might stabilize CtBP1 by preventing its degradation. Mutations in huntingtin protein might reduce the stability of CtBP1, which in turn would affect PA levels or change specific types of PA. CtBP1 is a redox/energy sensor combined with NAD(H) [153]; hence, it can be correlated with the synaptic energy state produced by PA. PA is a mandatory intermediate in the Kennedy pathway for the de novo synthesis of most phospholipids [154] and regulates proliferation through mammalian targets of rapamycin (mTOR) [155]. PA is also a signaling intermediate in the presynaptic and postsynaptic areas of neural networks. PA can be acquired from diacylglycerol (DG) by the catalysis of diacylglycerol kinases (DGKs), which are also used to complete the signal transmission of PKC, which is supervised by DG [156]. Research suggests that phosphatidic acid (PA) species may play a crucial role as active signal molecules, with distinct acyl chains compared to diacylglycerol (DG). This implies that the termination of DG-mediated signaling pathways does not necessarily indicate the presence of an active PA signal. In addition, a study employing microarray analysis reported an increase in diacylglycerol kinase (DGK) expression in the striatum of R/6-2 Huntington’s disease (HD) transgenic mice, providing valuable insights into the molecular mechanisms underlying HD [157]. Shing and colleagues investigated the impact of reducing mutant huntingtin (m-htt) proteins on brain lipids in a LacQ140 mouse model of HD using a systems biology approach [47] (Supplementary Table S1). Their study revealed temporary but noticeable changes in lipids abundant in myelin, suggesting potential white matter damage and regeneration in the brain, which may be linked to transcriptional changes in lipid metabolic enzymes. Lipid subclasses show significant concentration changes, including increases in glycerophospholipids, as well as significant reductions in the subclasses sphingomyelin (SM) and ceramide (Cer). Their findings further suggest that early and sustained reductions in M-Htt can prevent changes in the levels of select striatal proteins and most lipids, but a misfolded, degradation-resistant form of M-HTT hampers some benefits in the long term. Further insights into the metabolic changes associated with Huntington’s disease (HD) were gained through metabolic profiling in the R6/2 mouse model. Notably, this analysis revealed an increase in cholesterol synthesis precursors in the brain tissue of 6-week-old R6/1 mice [158]. Conversely, a decrease in the levels of specific amino acids, including alanine, aspartic acid, and N-acetyl aspartic acid (NAA), was observed in the striatum of late-symptomatic R6/2 mice [159]. A targeted lipidomics approach identified perturbations in the cholesterol pathways in both human post-mortem striatal and cortical cells in vivo as well as in brain tissue from HD mice (R6/2 mouse model overexpressing exon 1 of human HTT) [160]. This molecular dysfunction is biologically relevant because it results in reduced cholesterol biosynthesis in cultured human HD cells and significantly lower total cholesterol mass in brain-derived ST14A cells from HD mice and brain-derived cells from HD mice that express M-htt [50]. Emerging data suggest that glycosphingolipid dysfunction is one of the common primary factors among all the molecular pathways impacted by the mutation in HD [49,160,161,162]. Using both ultrastructural and lipidomic investigation, the potential relationship between glycosphingolipid modulator THI-induced [163] sphingolipid regulation and myelin structure in the R6/2 mouse striatum was evaluated [50]. The findings of that study demonstrated that administering THI to HD mice model prevented pathologically increased axon width and area, while maintaining myelin thickness and overall structure. Even though it is commonly known that HD primarily affects the caudate–putamen in terms of cell loss, hippocampal-dependent cognitive dysfunction has been found to occur early in HD patients [164]. Cognitive deficits, such as memory and spatial learning impairment, have been shown to occur before motor symptoms in HD [165,166,167]. The longitudinal spatial mapping of lipid metabolites in the hippocampi of HD transgenic mice by MALDI-MSI resulted in aberrant and/or adaptive changes to the lipid metabolism in the hippocampus of HD mice, which were observed prior to inclusions and symptoms developing. Early in the disease’s pre-symptomatic phase, patterns of alterations were seen in hippocampus neurons that included inclusion formation, axonal degradation, activation of the brain-derived neurotrophic factor (BDNF), hippocampal neurogenesis, synaptic dysfunction, and lipid homeostasis. These modifications evolved as the disease progressed over time and the patterns of alterations are associated with the important roles of synaptic plasticity and ER stress [51]. In a study carried out by Tshilenge et al., they sought to define the proteome of human HD patient-derived medium-spiny projection neurons (MSNs) from the striatum. Notably, lipid metabolism pathways were reported to be altered, and using quantitative image analysis, it was found that lipid droplets accumulated in the HD 72-MSN, suggesting a deficit in the turnover of lipids, possibly through lipophagy [168].

To identify potential plasma biomarkers, targeted lipidomics strategies relying on liquid chromatography/mass spectrometry (LC/MS) were employed to evaluate biochemical alterations in a pre-symptomatic HD sheep model [52]. The sheep model of HD exhibited a notable reduction in plasma sphingolipid and acylcarnitine levels. Most of the measured acylcarnitines (8 of 11) were significantly altered by genotype, with six showing reduced concentrations and two (hydroxybutyrylcarnitine (C3-DC(C4-OH)); glutaconylcarnitine (C5-1-DC)) showing increased concentrations. All but one (SM C20:2) of the fourteen sphingolipids (93%) quantified were significantly reduced in the HD sheep compared to the controls. The advancing neurodegeneration observed in HD involves a diminishing volume of both grey and white matter, as well as myelin degradation. This decline may be related to the decreased presence of sphingolipids in the bloodstream. The available evidence strongly indicates that various lipidomic changes occur early in the course of Huntington’s disease, potentially influencing the damage and advancement of the condition even prior to the onset of symptoms [52]. In a previous study that examined metabolic profiles in a transgenic sheep model of HD (OVT73) during the pre-symptomatic stages using LC/MS, the researchers identified a dysregulation in the circadian rhythmicity of metabolites, particularly phosphatidylcholines, in the pre-symptomatic sheep [169]. A ^1^H-NMR-based lipidomics study using CSF and serum samples from rats exhibiting HD symptoms revealed significant alterations in metabolites related to the mitochondrial respiratory chain, tricarboxylic acid (TCA) cycle, and lipids, indicating a malfunction in energy metabolism. Notably, the rats showed the re-amplification and transmission of 51 CAG repeat units, indicating that mitochondrial dysfunction occurs early in the progression of HD [53]. The enzyme glutaminase, localized to neuronal mitochondria, catalyzes the conversion of glutamine to glutamic acid. The interruption of the glutamate–glutamine cycle may result in impaired energy metabolism and mitochondrial respiration [159]. Furthermore, the notion that HD transgenic rats exhibit impaired oxidative energy metabolism is reinforced by the observed discrepancy in lactic acid levels between transgenic animals and their control counterparts. This difference in lactic acid levels suggests that transgenic rats may rely more heavily on anaerobic glycolysis, indicating a deficiency in oxidative energy production. In aerobic cellular respiration, pyruvate undergoes oxidation to form acetyl-CoA, which subsequently enters the citric acid cycle. Concurrently, reduced nicotinamide adenine dinucleotide (NAD+), generated during glycolytic reactions, is re-oxidized via the electron transport chain located in the mitochondria. However, a disruption in the citric acid cycle or the mitochondrial electron transport chain can impede the entry of pyruvate into the oxidative energy metabolism. Because reduced NAD^+^ must be re-oxidized to NAD^+^ to stabilize the state, another method of transferring electrons might be used wherein pyruvate is reduced to lactic acid through a reaction catalyzed by lactate dehydrogenase. Consequently, the accumulation of lactic acid, the end-product of anaerobic glycolysis, occurs, resulting in a compromised energy metabolism [170].

### 5.3. Human Samples

While central and functional alterations in the brain can serve as potential biomarkers for Huntington’s disease, it is important to acknowledge that the insights gained from animal studies often do not directly translate to humans, as highlighted in reference [171], and drawing clear conclusions from animals to humans poses significant challenges, as highlighted in [172]. Hence, to deepen our understanding of the disease, obtaining high-quality human tissue samples is of utmost importance. Analyzing the distinct lipidomic features of HD through clinical samples can substantially enhance our understanding of the disease’s pathological aspects. Indeed, these designated profiles can play a pivotal role in patient screening, as well as in monitoring the pharmacovigilance and efficacy of treatment modalities. This knowledge is also instrumental in achieving timely diagnoses, especially considering the considerable variability in clinical evaluation scores and the progression of HD. Studies examining postmortem cerebellum tissue from healthy individuals and those with HD revealed that alterations in membrane glycerophospholipids contribute to changes in GABA receptor affinity in HD [54]. Using HPLC and electrochemical detection, Ellison et al. found that greater alterations in ethanolamine and phosphoethanolamine are involved; both showed a reduced level in the metabolism of phospholipids in the striatum of HD patients [54]. A study conducted by Zarate et al. reported a comparable trend in the level of phosphoethanolamine in HD mouse brain by ^1^H-MRS, corroborating previous findings [30]. Additionally, research found that HD patients had lower levels of docosahexaenoic acid in their red blood cell membrane phospholipids compared to controls [173]. Moreover, HD patients exhibited a reduced response to niacin in the erythema test, which may be related to altered arachidonic acid release and prostaglandin D2 production, a regulator of niacin-induced vasodilation [46,174]. There is little information on the actual levels of specific glycerophospholipids and how these changes play a role in HD pathology.

The neurogenic activity in the subventricular zone (SVZ) shows that different disease states have different responses to the degeneration of neurons. In Parkinson’s and Alzheimer’s diseases, SVZ neurogenesis is suppressed [175]. In contrast, HD is distinguished by the gradual disappearance of fine neuron cells around the putamen and caudate nucleus (collectively referred to as the striatum), resulting in increased neural stem cell (NSC) activity in the SVZ [55].

In the postmortem brains of four Vonsattel grade III cases, high-resolution MALDI/IMS was performed on the center of the caudal part of the SVZ to study the lipidomic signature of the SVZ in HD patients [176]. The myelin layer in HD brain has a low sulfide and triacylglycerol (TAG) content. In the past, it has been reported that several lipid classes, especially TAG and sulfatides (STs), are enriched in the myelin sheath within the brain and spinal cord, which is neurologically normal [177]. However, in the lipidomic signature in the myelin sheath of HD patients, the number of STs is reduced by 40–60% [176].

STs are categorized as the least significant detectable lipids in the HD myelin sheath. In neurologically normal lamina IV, several lower STs are among the most abundant lipids. The low intensity of these species in HD is related to the apparent distribution of myelin-specific compartmentalization in MALDI images. In normal SVZ, three TAGs within the myelin sheath exhibit a comparable reduction of approximately 40% in HD, making them useful for distinguishing HD from normal cases. In normal SVZ (neurologically), TAG enrichment in the myelin sheath is definite, but there is no such pattern in HD. Sphingomyelins (SMs) are enriched in the myelin sheath of HD patients. Although the myelin sheath loses its strength due to the loss of TAG and STs, resulting in the loss of its spatial pattern, ectopic SM concentrations were observed in the lamina IV of the HD brain. Six sphingolipid samples were 1.5 to 2.5 times more enriched in HD than in normal samples. When the area under the ROC curve is less than 0.25, very strong discriminative statistics are obtained. MALDI images show an intense concentration of SMs in the lamina IV of HD patients, which is not present in normal brain; these findings are in agreement with the findings that SMs are available in the SVZ, not only in the myelin sheath [177].

Phosphatidylinositol (PI) is reduced in the ependyma of HD patients. An investigation of lipidomic signatures in normal human SVZ showed the segmentation of PI in the interventricular septum, which is believed to reflect its role in ciliary body generation and interventricular septal polarization [177]. This study showed that the loss of PI in the membrane protein of HD ependyma is significant; 10 of the phospholipids studied steadily decreased by 30–50%. A notable alteration in the positioning of ependymal cells is evident in the MALDI images, indicating a reversal of their normal orientation. Furthermore, a decrease in signal intensity is observed in the parenchyma located beneath the caudate nucleus, suggesting changes in the molecular composition of this region.

The detection of increased SMs in the HD myelin layer is significant when considering the potential function of these species in the enhanced proliferation observed in the SVZ of HD patients [55]. Sphingomyelin is a common sphingolipid, prevalent in cell membranes, especially in microdomains like caveolae, clusters of lipids, and other structures that play a role in transmembrane signaling [81]. Ceramide is an effective sphingolipid metabolite that regulates cellular processes and is essential for the sphingolipid signaling network [178]. In addition, sphingolipids originate from ceramide, which regulates the signaling paths related to neural differentiation, cell cycle arrest, and apoptosis. It is thought that the greater abundance of SMs in the myelin sheath of HD patients might be due to ST degeneration. Taking into consideration the cellular function of SMs, the lipids mentioned can enhance the stimulation derived from the adjacent degenerate caudate tail, thereby promoting neurogenesis and cell proliferation in the SVZ.

In HD, some of the most studied lipids include lipid peroxidation end products [179]. In one such study, 8-hydroxy-2′-deoxyguanosine and other DNA oxidation markers show that no increase or change in lipid peroxidation was observed (through a nonspecific marker of lipid peroxidation—thiobarbituric acid-reactive substances (TBARS)) in HD [180]. In contrast, other studies showed that the HD lipid peroxidation markers malondialdehyde and F2-isoprostaglandin were elevated [179]. In a targeted lipidomics study by Valenza et al., it was shown that the activity of the cholesterol biosynthetic pathway is altered in HD cells, mice, and human subjects [48]. This impairment and subsequent drop in the end product (i.e., cholesterol level), reduced total cholesterol mass, and reduced transcription of important genes involved in cholesterol biosynthesis are caused by a mutant huntingtin-dependent reduction in the quantity of the active form of the SREBP transcription factor translocating to the nucleus. As part of a comprehensive effort to identify novel biomarkers for HD, Underwood et al. conducted a metabolic profiling study using gas chromatography–time-of-flight–mass spectrometry (GC-TOF-MS). This approach involved analyzing blood samples from human HD patients, as well as a transgenic mouse model of the disease. The goal of this hypothesis-generating study was to uncover potential biomarkers that could facilitate the diagnosis, monitoring, and treatment of HD [56]. Notably, both transgenic mice and their wild-type littermates exhibited unique alterations in their metabolic profiles. Furthermore, a similar trend was observed in human patients and control subjects, characterized by significant modifications in several markers of fatty acid β-oxidation, including glycerol and malonate.

A targeted mass spectrometry approach was employed to investigate alterations in sphingolipid species within the brains of clinically advanced HD patients. This analysis utilized post-mortem brain tissue samples from five brain regions implicated in HD pathology, obtained from 13 HD patients and 13 control individuals [57]. For the first time, an increased abundance of long-chain and a corresponding decreased abundance of very-long-chain ceramides, sphingomyelins, and lactosylceramides were identified in the caudate of clinically advanced HD patients, whilst the white and grey frontal cortex were spared. A recent investigation employed matrix-assisted laser desorption/ionization imaging mass spectrometry (MALDI-IMS) to generate a spatially resolved and comprehensive lipidomic atlas of the caudate nucleus (CN). This study analyzed post-mortem human tissue samples obtained from 10 neurologically normal individuals and 13 subjects with Huntington’s disease (HD). The grey and white matter components of the CN were shown to exhibit lipidomic specialization, and these characteristics were substantially maintained between participants. The CN specimens from the HD cases spanning a range of neuropathological grades showed a lower focal abundance of neuroprotective docosahexaenoic and adrenic acids, several cardiolipins, the ganglioside GM1, and glycerophospholipids with long polyunsaturated fatty acyls, even though most lipid species were highly conserved in HD compared to the age-matched controls.

A greater focal abundance of various sphingomyelins and glycerophospholipids with shorter monosaturated fatty acyls was seen in HD patients [3]. In a recent multi-omics study carried out by Paryani et al. [58], the lipidomic analysis of HD and control brains revealed that several lipid species, including ceramides and very-long-chain fatty acids, are associated with HD severity. The unfolded protein response pathway was implicated in the consensus gene signature that was found through the integration of lipidomics and bulk transcriptomics. This gene signature corresponds with both HD grade and HD lipidomic abnormalities. Further, studies have shown that astrocytes in HD display region-specific and state-dependent responses to HD pathology in human brain samples. Using cutting-edge mass spectrometry techniques (DI/LC-MS/MS), Graham et al. [59] accurately identified and measured 185 metabolites in postmortem frontal lobe and striatum tissues—the region most severely impacted in HD patients—and compared them to healthy controls, revealing valuable insights into HD’s metabolic landscape (Supplementary Table S2).

This study reveals a connection between altered phospholipid metabolism and HD pathology. Further, significant differences in the lipid profile of two brain regions were observed, which might be explained by extensive nerve loss in the striatum. Di Pardo et al. [162] found variations in sphingosine-1-phosphate (S1P)-metabolizing enzymes in both preclinical HD models and patients with HD compared to healthy controls. Specifically, the PM striatum and cortex of patients with HD, unlike the controls, exhibited up-regulated S1P lyase 1 and down-regulated sphingosine kinase 1 levels, indicating a perturbation in sphingolipid metabolism in patients with HD [181]. Furthermore, human studies have investigated cholesterol- and cholesteryl ester (CE)-level disturbances in HD [60]. In particular, the caudate and putamen of patients with HD have been shown to contain elevated levels of CE, which, in turn, reduces cholesterol accumulation as a counteracting mechanism [60]. In a recent study using single-nuclei RNA sequencing, Reyes-Ortiz et al. analyzed astrocytes from HD patients’ iPSCs and R6/2 HD mice, revealing impaired maturation and glutamate signaling [182]. In this study, it was found that astrocyte dysregulation in R6/2 mice worsened from 8 to 12 weeks, overlapping across brain regions and with human data. Cortical astrocytes at 8 weeks showed distinct SP1 signaling and lipid metabolism alterations, overlapping across brain regions and with human data [182].

The collection of serum/plasma samples for lipidomic analysis is easy and minimally invasive compared to collecting other study materials, such as antemortem brain tissue or CSF, either for longitudinal studies or for use in preclinical and clinical settings. However, only a few studies have investigated the lipidomics of HD in blood products from HD patients. In a recent study utilizing ^1^H NMR spectroscopy, Chang et al. investigated the alternations of 112 lipoprotein subfractions and components and their associated levels of metabolites as biomarkers of HD in the plasma of nine presymptomatic (preHD), twenty normal controls, and twenty symptomatic (sympHD) HD patients [61]. Significant concentration changes in 30 lipoprotein subfractions and components in all HD patients were reported, including significant decreases in HDL3-FC, HDL4-CH, HDL4-ApoA1, and HDL4-FC [61]. A comparative analysis of plasma lipid profiles revealed that individuals diagnosed with pre-HD and sympHD exhibited decreased levels of total cholesterol (CH), apolipoprotein B (Apo)B, apolipoprotein B particle number (PN), and low-density lipoprotein (LDL) constituents. Furthermore, these patients displayed reduced levels of specific LDL and high-density lipoprotein (HDL) components compared to the controls. The components of LDL3 displayed lower levels in sympHD patients compared with the controls, whereas the components of very-low-density lipoprotein (VLDL5) were higher in the sympHD patients compared to the controls. The component levels of HDL4 and VLDL5 were correlated with motor assessment, independence scale, and functional capacity score on the Unified HD Rating Scale (UHDRS). The findings from this study highlight the role played by lipids and apolipoproteins in HD pathogenesis and suggest that components of VLDL5, LDL3, LDL4, LDL5, and HDL4 may function as biomarkers for HD diagnosis and disease progression. There is evidence to suggest that HDL contains anti-apoptotic, antioxidant, anti-thrombotic, and anti-inflammatory properties [183]. Mastrokolias et al. investigated metabolic and lipid pathway disorders, as well as serum metabolic markers, to monitor the status and progression of HD [62]. In that study, using targeted mass spectrometry-based metabolomics, serum metabolite levels in HD patients were measured and a linear model based on UHDRS scores that linked 10 metabolites to HD severity was generated. These metabolites included eight phosphatidylcholines and two amino acids (threonine and serine). Comparing these findings with untargeted metabolomics/lipidomics studies revealed a strong positive correlation, validating the results and demonstrating the benefits of combining data from different platforms for comprehensive metabolic analysis in future research [184,185]. The results align with a previous study where phosphatidylcholine levels decreased in lipid extracts from the precortical area of HD rats treated with mitochondrial toxin 3-nitropropionic acid (3-NP) [186]. Phosphatidylcholine, a crucial membrane phospholipid, plays a significant role in cellular fate and neuronal differentiation [187]. Prior research shows that consuming choline-rich foods effectively replaces phosphatidylcholine, potentially supporting acetylcholine synthesis in the brain [188]. Notably, Mastrokolias et al. [62] reported a negative correlation between eight phosphatidylcholine lipid groups (PC aa C38:6, PC aa C36:0, PC ae C38:0, PC aa C38:0, PC ae C38:6, PC ae C42:0, PC aa C36:5, and PC ae C36:0) and disease progression, suggesting altered lipid metabolism in neurodegenerative diseases [37,189]. This indeed implies that phosphatidylcholine supplementation could be a potential therapeutic approach. A study conducted by Cheng et al. [190] utilized a comprehensive panel of features from carnitines and phosphatidylcholine species to distinguish between individuals with HD and healthy controls. This global screening approach was applied to plasma samples from 15 HD patients and 17 controls. Furthermore, the researchers quantified 184 related metabolites, including carnitine, amino acid, and phosphatidylcholine species, in a larger cohort comprising 29 HD patients, 9 pre-symptomatic HD carriers, and 44 controls. This analysis revealed significant alterations in metabolic profiles, including the up-regulation of glycine and the down-regulation of nine metabolites/lipids. These findings provide novel insights into the involvement of disturbed metabolism in HD pathogenesis and highlight the potential for developing innovative treatment strategies.

Despite the potential discomfort associated with cerebrospinal fluid (CSF) collection, research on neurodegenerative diseases affecting the brain is greatly improved by studies involving CSF due to its proximity to the central nervous system. Notably, over 450 metabolites have been identified and quantified in human CSF [191], some of which have been the subject of study in HD [192] and other neurological conditions, such as multiple sclerosis [193], Alzheimer’s disease [106], and Parkinson’s disease [194]. A study conducted by Herman et al. [181] revealed significant disruptions in phospholipid metabolism—in particular, significant changes in the level of glycerophospholipids and sphingolipids—when comparing the cerebrospinal fluid (CSF) of HD patients with that of healthy control subjects [192].

A recent cross-sectional analysis examined the plasma and cerebrospinal fluid (CSF) profiles of individuals with HD. The results showed significantly elevated plasma levels of arginine, citrulline, and glycine, accompanied by decreased levels of total D-serine, cholesterol esters, diacylglycerides, triacylglycerides, phosphatidylcholines, phosphatidylethanolamines, and sphingomyelins. In contrast, the CSF analysis revealed that disease progression was linked to increased levels of NAD+, arginine, saturated long-chain free fatty acids, diacylglycerides, triacylglycerides, and sphingomyelins, although these changes were only nominally significant [63].

The in silico analysis of pre-symptomatic and symptomatic HD patients showed that the deregulated pathways include metabolic pathways of various amino acids, glutathione metabolism, longevity, autophagy, and mitophagy. Peripheral biomarkers play a vital role in tracking the efficacy of treatments, monitoring disease progression, and preventing symptoms.

For example, total htt protein was measured in the saliva of 98 patients with manifest HD, gene-positive premanifest HD, and control subjects (matched for sex and age) [195]. To further elucidate the complex biological mechanisms at play, additional saliva tests were conducted using standardized ELISA methods. These tests focused on a panel of key biomarkers associated with inflammation, including C-reactive protein, cortisol, interleukin-1β, and interleukin-6. By examining these biomarkers, researchers aimed to gain a more comprehensive understanding of the underlying inflammatory processes. The measurement of saliva proteins, especially htt, has potential as an important noninvasive biomarker for the onset of HD symptoms and disease progression. However, no lipidomics data were found in the saliva of HD patients. Nevertheless, the potential of saliva as a diagnostic tool should not be overlooked; its convenience and accessibility make it an attractive medium for uncovering new biomarkers, which could lead to significant advancements in various fields of research. Human saliva is a complex biofluid that, in addition to its high water content, comprises a multitude of biomolecules, including electrolytes, proteins, glycoproteins, nucleic acids, hormones, lipids, and metabolites [196,197]. These biomolecules have the potential to serve as biomarkers for detecting and monitoring various disease states The great potential of saliva for the investigation of HD biomarkers through various omics approaches has been widely demonstrated by several investigators [195,198,199,200,201,202,203,204]; however, to date, no lipidomics investigation utilizing saliva samples from HD patients has ever been reported. By expanding our understanding of the markers present in saliva, we may be able to build non-invasive HD management solutions that are more widely available and reasonably affordable than current approaches.

## 6. Future Directions and Clinical Implications

Lipidomics—the study of lipid metabolism at the global, or “-omics”, level—is a rapidly emerging field with great potential to uncover the molecular mechanisms underlying various diseases. The past decade has witnessed significant advancements in this field, characterized by the development of novel methodologies, enhanced sensitivity of measurements, and innovations in computational analysis and data interpretation. A more comprehensive understanding of global perturbations in lipid pathways in complex diseases such as HD and upon treatment with drugs could provide valuable insights into mechanisms of disease, drug effects, and variation in drug response, and potentially provide needed prognostic, diagnostic, and surrogate biomarkers. To fully harness the potential of saliva as a diagnostic tool, it is essential to address and overcome the existing limitations that currently hinder its effectiveness. Future research must concentrate on emerging high-throughput products that can enrich lipids from blood without contaminating it, on developing novel isolation techniques that can capture lipids specific to CNS cell types, and on creating lipidomic platforms that are clinically applicable to overcome these limitations. One of the main concerns regarding the findings summarized in this review is that most lipid classes have not been consistently found to be associated with HD. Variables such as sex, age, HD etiology, specific DNA polymorphisms, and the microbiome may have influenced the findings. Thus, a proper stratification of HD patients is necessary to understand the biological implications of the lipid changes observed. Additionally, more accurate descriptions of the lipid profiles of plasma, CSF, and/or fibroblasts from HD patients will help to classify patients more accurately. Furthermore, research should concentrate on improving our knowledge of the function of lipids in HD and developing methods to modify lipids for therapeutic purposes. Once such knowledge on the association between lipid metabolism and the pathophysiology of HD is gained, there is huge potential to find new novel treatment modalities. The promise of lipids in HD research may be accomplished with the correct blend of scientific understanding and technical advancements. Finally, considering the inherent molecular and clinical heterogeneities in HD, an integrated omics approach incorporating lipidomics is essential for identifying accurate and robust biomarkers through the investigation of the cross-talk between different biological systems, and will aid in revealing alterations in the central dogma [205]. Even though the field of brain lipidomics is still in its infancy, we firmly believe that the extensive amounts of valuable data generated through lipidomics analyses will play a pivotal role in advancing our understanding of brain function and disease. This, in turn, will greatly benefit future clinical brain research.

## Figures and Tables

**Table 1:** List of studies published on lipidomic screening in HD.

Model Organism	Investigated Lipid Species	Description	Reference
Cell culture model	Sphingolipids, fatty acids, and steroids	We present PIUMet, a network-based approach for untargeted metabolomics analysis that reconstructs molecular pathways and identifies key components without requiring individual metabolite identification.	[40]
Transgenic Drosophila model of HD.	Intracellular lipid droplets	The association between Huntington protein (Htt) neuronal expression and the destabilization of metabolism, affecting weight, nutrients, and lipids was reported.	[41]
Cell culture model (COS-1, MCF-7 and X57 cells)	Phospholipids	Polyglutamine expansion disrupts N- Htt localization. No binding differences reported, but results are limited due to potential varying forms of M-htt. M-Htt may self-aggregate or bind to interacting proteins, causing mislocalization.	[42]
Allelic panel of heterozygous CAG knock-in mouse embryonic stem	Lipids, steroids, and cholesterols	Alterations in mRNA levels that regulate cholesterol, sterol, and phospholipid metabolism were observed.	[43]
Hdh^140Q/140Q^ mice or wild-type mice; primary human fibroblasts (normal and HD); clonal striatal X57 cells	Phospholipids	Htt interacts with membranes through specific phospholipid associations and M-htt may disrupt membrane trafficking and signaling.	[44]
Hdh^140Q/140Q^ mice or wild-type mice	Glycerophospholipid	Increases in species of lyso-PA (LPA) were detected in striatum of Hdh^140Q/140Q^ compared to WT.	[45]
Patients with advanced (stage III) HD and gender- and sex-matched normal individuals with no history of HD or any other major neurological disorder	Fatty acid metabolism	HD may be associated with an abnormality of neuronal membrane fatty acid metabolism, possibly as a consequence of an as-yet-unidentified action of htt.	[46]
Knock-in Q175/Q7 HD and Q7/Q7 mice	Phosphatidylcholine, Acyl Carnitine, Ceramides, Triglyceride, Acyl Carnitine, Triglyceride	Lipidomic analysis showed that the presence of the HD mutation conferred age-dependent disruption of localization of synaptic proteins and lipids important for synaptic function.	[4]
LacQ140 mouse model of HD	Phosphatidylcholine, Acyl Carnitine, Ceramides, Triglyceride, Acyl Carnitine, Triglyceride	Early and sustained reduction in mHtt can prevent changes in levels of select striatal proteins and most lipids, but a misfolded, degradation-resistant form of mHTT hampers some benefits in the long term.	[47]
HD mice and brain-derived ST14A cells	Cholesterol	The cholesterol biosynthetic pathway is impaired in HD cells, mice, and human subjects.	[48]
R6/1 transgenic mouse, as well as postmortem caudate from human HD patients	Glycosphingolipid, Glycolipids, Ganglioside	Novel disruptions in glycolipid and ganglioside metabolic pathways in the pathology of HD have been observed.	[49]
R6/1 transgenic mouse model of HD	Glycosphingolipid	Glycosphingolipid metabolism in HD is druggable; thus, this pathway may represent a new approach for the treatment of the disease.	[50]
Transgenic R6/1 mouse model of HD	Phosphatidylethanolamine Phosphatidylinositol, Phosphatidic acid, Ganglioside, Sphingolipids, Ceramide, Cardiolipins	Demonstrated aberrant and/or adaptive changes to lipid metabolism in the hippocampus of HD mice were detected before inclusions and symptoms developed.	[51]
Transgenic sheep model of HD	Acylcarnitines, Sphingolipids, Glycerophospholipids	A biomarker panel mainly consisting of lipids to define the stage or track the progression of disease would be invaluable for measuring therapeutic effects in HD.	[52]
Transgenic rat model of HD	Lipid moieties in both serum and CSF NMR spectra	Both serum and CSF showed lower level of lipid concentration in TG animals.	[53]
Postmortem brain samples from patients with AD and HD	Phosphoethanolamine (PEA) and Ethanolamine (EA)	In HD, concentrations of PEA were significantly reduced by 76% in the caudate, 53% in the putamen, and 48% in the nucleus accumbens.	[54]
Postmortem control and HD human brain tissue	Sphingomyelins (SM)	Elevated SMs in HD myelin may drive enhanced cell proliferation in the SVZ.	[55]
Serum samples obtained from both HD patients/controls and transgenic mouse model of HD/wild-types littermates	Fatty acid breakdown (including glycerol and malonate)	Metabolic/lipidomic profiling identified HD biomarkers in human and mouse serum via GC-TOF-MS.	[56]
Post-mortem brain tissue from five brain regions implicated in HD (control *n* = 13; HD *n* = 13)	Sphingolipid	Caudate, putamen, and cerebellum had distinct sphingolipid changes in HD brain whilst the white and grey frontal cortex were spared.	[57]
Post-mortem brain tissue from HD brain and healthy controls	Global lipidomic profiling	CAG repeat length-correlated genes were enriched in astrocytes and lipidomic signatures, implicating poly-unsaturated fatty acids in neuronal vulnerability.	[58]
Post-mortem brain tissue from HD brain and healthy controls (frontal lobe, striatum)	Global lipidomic profiling	Acylcarnitine and phospholipid metabolism emerge as critical targets for HD.	[59]
Post-mortem tissue from HD subjects and age- and sex-matched controls	Cholesteryl esters (CE)	The striatal region-specific differences in CE profiles indicate functional subareas of lipid disturbance in HD.	[60]
The plasma of normal controls, symptomatic (sympHD) and presymptomatic (preHD) HD patients.	Lipoprotein subfraction	VLDL5, LDL3-5, and HDL4 components show promise as HD biomarkers, highlighting lipids’ role in disease pathogenesis.	[61]
HD patient and control serum samples	Phosphatidylcholines	Phosphatidylcholine metabolism is deregulated in HD blood and these metabolite alterations are associated with specific gene expression changes.	[62]
Plasma and CSF profiles from individuals with HD and controls	Cholesterol esters, diacylglycerides, triacylglycerides, phosphatidylcholines, phosphatidylethanolamines, and sphingomyelins	Worsening disease was associated with nominally significant increases in saturated long-chain free fatty acids, diacylglycerides, triacyl glycerides, and sphingomyelins.	[63]

**Table 2:** Bioinformatic and computational tools available for lipidomics. Available bioinformatic tools developed for (1) data processing and lipid identification, (2) statistical data analysis, (3) pathway analysis, and (4) lipid modelling in systems and biophysical contexts.

Technique	Mass Spectrometry (MS)	Nuclear Magnetic Resonance (NMR)
Fields used in	Diverse use (proteomics, metabolomics, lipidomics, drug discovery, toxicology)	Diverse use (proteomics, metabolomics, lipidomics, drug discovery, toxicology)
Detection mechanism	Chemical compounds are converted into gas-phase molecules, and their mass-to-charge (*m*/*z*) ratio is measured	Electromagnetic radiation sources can be tuned to different frequencies; therefore, NMR acquires spectra from different kinds of nuclei
Compatible	Solid/gas/liquid	Solid/liquid
Sensitivity	High (nanogram to picogram)	Low (milligram to nanogram)
Selectivity	Both targeted (selective) and non-targeted (non-selective) assays can be performed	Non-selective analysis
Reproducibility	Moderate to high, depending on the sample clean-up and biochemical properties of analyte of interest	High
Sample preparation	Time-consuming and depends on the sample matrix; liquid/liquid/solid-phase extraction or chemical derivatization can be used	Compared to MS, NMR sample preparation is minimal
Sample volume	Biological fluid: 5–500 µL (depends on the assay) Cells: 3–10 million Tissue: 10–25 mg	Biological fluid: 50–500 µL (depends on the assay) Cells: 15–25 million Tissue: 25 mg to check
Sample matrix	Tissue, cells, serum, saliva, tears, hair, ear wax, CSF, plasma, urine, whole blood	Tissue, cells, serum, saliva, tears, hair, ear wax, CSF, plasma, urine, whole blood
No. of identified metabolites/lipidomics	100 to more than 1000 in a single experiment	40–200 depending on spectral resolution
Quantitation	Qualitative and quantitative analysis can be performed; need isotope-labeled standards and calibration curves for each analyte for absolute quantitation	Absolute quantitation requires a standard of known concentration
Advantages	GC-MS: relatively inexpensive, modest sample size, great sensitivity, a large body of software available and databases for metabolite ID; LC-MS: detects most organic and inorganic molecules, minimal sample size required, direct injection can be possible, has the potential to detect largest portion of the metabolome and lipidome	Quantitative (^1^H NMR), non-destructive, fast, requires no derivatization, detects all organic classes, allows ID of novel metabolites, robust, large body of software and database available for metabolite ID
Disadvantages	GC-MS: time-consuming, novel ID is difficult, longer run time; LC-MS: time-consuming and longer run time, depends on the type of LC used	Less sensitive than MS and expensive to maintain

**Table 3:** Comparison of main analytical platforms utilized in the field of lipidomics of Huntington’s disease.

	Data Type	Description	Link to Access
MetaboAnalyst	NMR and MS data	Platform for metabolomics including lipidomics providing comprehensive data pre-processing, statistical analysis, functional analysis, meta-analysis, and integrative analysis with other omics data.	https://metaboanalyst.ca/
GNPs	MS data	GNPS is a web-based mass spectrometry ecosystem that aims to be an open access knowledge base for community-wide organization and the sharing of raw, processed, or annotated fragmentation mass spectrometry data, including lipidomics data.	https://gnps.ucsd.edu/ProteoSAFe/static/gnps-splash.jsp
lipidr	NMR and MS data	Data mining and analysis of lipidomics datasets in R.	https://www.lipidr.org/
MetaboLights	NMR and MS data	Database for metabolomics, including lipidomics, providing metabolite structures, their biological information, and reference spectra. MetaboLights is the recommended metabolomics repository for a number of leading journals.	https://www.ebi.ac.uk/metabolights/
LIPID MAPS	MS data	LIPID MAPS (LIPID Metabolites and Pathways Strategy) provides a systematic and standardized approach to organizing lipid structural and biochemical data.	https://www.lipidmaps.org/
Metabolomics Workbench	NMR and MS data	A public repository for metabolomics, including lipidomics, metadata and experimental data spanning various species and experimental platforms, metabolite standards, metabolite structures, protocols, tutorials, training materials and other educational resources.	https://www.metabolomicsworkbench.org/
LipidSig	NMR and MS data	LipidSig is the first web-based platform which integrates a comprehensive analysis for the streamlined data mining of lipidomic datasets.	https://lipidsig.bioinfomics.org/
HMDB	NMR and MS data	The Human Metabolome Database (HMDB) is currently the most complete and comprehensive curated collection of human metabolites (including lipids) and human metabolism data in the world. It contains records for more than 2180 endogenous metabolites with information gathered from thousands of books, journal articles, and electronic databases. In addition to its comprehensive literature-derived data, the HMDB also contains an extensive collection of experimental metabolite concentration data.	https://hmdb.ca/
Lipid Bank	NMR and MS data	“LipidBank” is an open, public, free database of natural lipids, including fatty acids, glycerolipids, sphingolipids, steroids, and various vitamins. The database contains more than 6000 unique molecular structures, their lipid names, spectral information, and most importantly, literature information.	https://lipidbank.jp/help/about.html
LipidSearch	MS data	LipidSearch is a software for processing data from direct infusion and LC-MS high-resolution mass spectrometry-based lipidomics workflows.	https://lcms.labrulez.com/paper/7946

## Data Availability

Not applicable.

## Supplementary Materials

The following supporting information can be downloaded at https://www.mdpi.com/article/10.3390/metabo15010010/s1, Table S1: Sphingomyelin (SM) and ceramide (Cer) showing significant concentration change in HD reported by Shing et al. Table S2: Lipid species detected in HD mouse brain reported by a study Graham et al.
