# Supplementary material for: Lipidomics of Huntington’s Disease: A Comprehensive Review of Current Status and Future Directions
Source: Metabolites. 2025 Jan 2;15(1):10. doi: 10.3390/metabo15010010 (PMC11766911; doi:10.3390/metabo15010010)
Supplement: Supplementary file 1 [file metabolites-15-00010-s001.zip › metabolites-3318972-supplementary.pdf]

Supplementary Table S1: Sphingomyelin (SM) and ceramide (Cer) showing significant concentration change in HD reported by Shing et al

Cer(d18:1_18:0)+HCOO	SM(d36:2)+HCOO	SM(d34:1)+H	SM(d42:1)+HCOO
Cer(d18:2_18:0)+HCOO	SM(d38:1)+H	SM(d34:1)+HCOO	SM(d42:2)+H
Cer(d18:2_18:0)+H	SM(d38:1)+HCOO	SM(d36:1)+H	SM(d42:2)+HCOO
Cer(d18:1_24:1)+HCOO	SM(d18:2_20:2)+H	SM(d36:1)+HCOO	SM(d44:5)+H
Cer(t18:0_18:0)+H-H2O	SM(d40:1)+H	SM(d36:2)+H	SM(t39:1)+H
Cer(t18:1_18:0)+H-H2O	SM(d40:1)+HCOO	SM(d41:1)+HCOO	SM(t39:2)+H
CerPE(d18:1_24:5)+H-H2O	SM(d40:2)+H	SM(d41:5)+H	SM(t18:0_22:2)+H
SM(d18:1_23:0)+H	SM(d18:1_24:0)+H		

Supplementary Table S2: Lipid species detected in HD mouse brain reported by a study Graham et al

C0	PC aa C42:2	C12:1	PC ae C38:5	Leucine
C2	PC aa C42:4	C14	Lysine	lysoPC a C14:0
C3	PC aa C42:5	C14:1	Methionine	lysoPC a C16:0
C3-DC (C4-OH)	PC aa C42:6	C14:1-OH	Ornithine	lysoPC a C16:1
C3-OH	PC ae C30:0	C14:2	Phenylalanine	lysoPC a C17:0
C3:1	PC ae C30:1	C14:2-OH	Proline	lysoPC a C18:0
C4	PC ae C30:2	C16	Serine	lysoPC a C18:1
C4:1	PC ae C32:1	C16-OH	Threonine	lysoPC a C18:2
C5	PC ae C32:2	C16:1	Tryptophan	lysoPC a C20:3
C5-DC (C6-OH)	PC ae C34:0	C16:1-OH	Tyrosine	lysoPC a C20:4
C5-M-DC	PC ae C34:1	C16:2	Valine	lysoPC a C24:0
C5-OH (C3-DC-M)	PC ae C34:2	C16:2-OH	Asymmetric dimethylarginine	lysoPC a C26:0
C5:1	PC ae C34:3	C18	alpha-aminoadipic acid	lysoPC a C26:1
C5:1-DC	PC ae C36:0	C18:1	Carnosine	lysoPC a C28:0
C6 (C4:1-DC)	PC ae C36:1	C18:1-OH	Creatinine	lysoPC a C28:1

C6:1	PC ae C36:2	C18:2	L-Dopa	PC aa C24:0
C7-DC	PC ae C36:3	Alanine	Histamine	PC aa C26:0
C8	PC ae C36:4	Asparagine	Kynurenine	PC aa C28:1
C9	PC ae C36:5	Citrulline	Putrescine	PC aa C30:0
C10	PC ae C38:0	Glutamine	Sarcosine	PC aa C32:0
C10:1	PC ae C38:1	Glutamate	Symmetric dimethylarginine	PC aa C32:1
C10:2	PC ae C38:2	Glycine	Spermidine	PC aa C32:2
C12	PC ae C38:3	Histidine	Spermine	PC aa C32:3
C12-DC	PC ae C38:4	Isoleucine	trans-hydroxy-Proline	PC aa C34:1
PC ae C38:6	SM C18:0	PC ae C42:2	PC aa C36:6	PC aa C34:2
PC ae C40:1	SM C18:1	PC ae C42:3	PC aa C38:0	PC aa C34:3
PC ae C40:2	SM C20:2	PC ae C42:4	PC aa C38:3	PC aa C34:4
PC ae C40:3	SM C24:0	PC ae C42:5	PC aa C38:4	PC aa C36:0
PC ae C40:4	SM C24:1	PC ae C44:3	PC aa C38:5	PC aa C36:1
PC ae C40:5	SM C26:0	PC ae C44:4	PC aa C38:6	PC aa C36:2
PC ae C40:6	SM C26:1	PC ae C44:5	PC aa C40:1	PC aa C36:3
PC ae C42:0	H1	PC ae C44:6	PC aa C40:2	PC aa C36:4
PC ae C42:1	PC aa C40:5	SM (OH) C14:1	PC aa C40:3	PC aa C36:5
SM (OH) C22:2	PC aa C40:6	SM (OH) C16:1	PC aa C40:4	SM C16:1
SM (OH) C24:1	PC aa C42:0	SM (OH) C22:1	SM C16:0	PC aa C42:1
